# Special Considerations in Pediatric Inflammatory Bowel Disease Pathology

**DOI:** 10.3390/diagnostics15070831

**Published:** 2025-03-25

**Authors:** Alicia R. Andrews, Juan Putra

**Affiliations:** 1 Department of Pathology and Laboratory Medicine, University of Saskatchewan, Saskatoon, SK S7N 0W8, Canada; alicia.andrews@saskhealthauthority.ca; 2 Department of Pathology, Boston Children’s Hospital and Harvard Medical School, Boston, MA 02115, USA

**Keywords:** Crohn disease, ulcerative colitis, lymphocytic esophagitis, focally enhanced gastritis, PSC-IBD, monogenic

## Abstract

Inflammatory bowel disease (IBD) in the pediatric population presents distinct characteristics compared to adult cases. Pathology plays a critical role in its diagnosis, and this review underscores key considerations in the pathologic evaluation of pediatric IBD. Recognizing inflammatory patterns in the upper gastrointestinal tract can improve disease classification and aid in diagnosing IBD in certain scenarios, such as isolated upper gastrointestinal or small bowel involvement. Additionally, familiarity with distinctive subtypes, including IBD associated with primary sclerosing cholangitis and monogenic forms of IBD, supports early comorbidity detection, enhances patient management, and improves prognostication.

## 1. Introduction

Inflammatory bowel disease (IBD) is a chronic immune-mediated disorder of the gastrointestinal (GI) tract, characterized by alternating periods of flares and remission. Its development involves a complex interaction among host genetics, microbiome, immune system, and environmental factors [1]. A recent systematic review by Kuenzig et al. highlighted the global rise in the incidence and prevalence of pediatric IBD, with the highest rates reported in Northern Europe and North America and the lowest in Southern Europe, Asia, and the Middle East [2].

What distinguishes IBD in the pediatric population from adult-onset cases? Pediatric IBD is often diagnosed earlier in the disease course, before the development of complications. For example, children are more likely to present with Crohn disease (CD) prior to the onset of fibrostenotic or penetrating complications [2]. However, pediatric-onset IBD has been associated with a more extensive and severe disease trajectory [3,4,5]. This is due to a longer lifetime risk of accumulating inflammatory damage and its occurrence in individuals with the highest genetic and environmental susceptibility to IBD. Ultimately, these factors may lead to more severe disease activity over time. Additionally, pediatric IBD presents unique challenges, including the transition of care to adult services, psychosocial and financial burdens, and the substantial cost of managing younger patients, which often exceeds that of older individuals due to their lifelong need for healthcare [2].

This review highlights key considerations in the pathologic evaluation of pediatric IBD, focusing on disease classification, upper GI involvement, distinctive phenotypic presentations, such as IBD associated with primary sclerosing cholangitis, and monogenic forms of the disease. Additionally, it discusses important mimickers that should be considered during a pathologic assessment.

## 2. Pediatric Inflammatory Bowel Disease (IBD) Classification

The diagnosis of pediatric IBD is typically established through a comprehensive approach that includes clinical history, physical examination, laboratory tests, upper endoscopy, ileocolonoscopy, histologic evaluation, and small bowel imaging. Other potential diagnoses, particularly infections, should be carefully excluded. Once diagnosed, pediatric IBD is generally classified into ulcerative colitis (UC), CD, or IBD-unclassified (IBD-U). Accurate classification is essential for guiding the treatment and predicting disease outcomes. However, classification is not always straightforward and requires recognizing the typical features of UC and CD, identifying atypical phenotypes that still align with these diagnoses, and understanding factors that may preclude a definitive classification [6].

UC is characterized by continuous mucosal inflammation of the colon, originating distally from the rectum. It typically does not involve the small intestine, except in cases of backwash ileitis, and non-necrotizing granulomas are absent on biopsy. In 2014, the revised Porto criteria expanded the classification of UC to include typical and atypical forms [6]. Atypical UC encompasses cases with features that deviate from the classic UC, but occur frequently enough to rule out a CD diagnosis. These features include macroscopic rectal sparing, patchy disease or lack of chronicity in biopsies, left-sided colitis with a cecal patch (periappendiceal inflammation), upper GI involvement, and acute severe presentations that resemble CD.

Key features of CD include skip lesions, well-formed non-necrotizing granulomas distant from ruptured crypts, and characteristic macroscopic findings in the upper GI tract (e.g., serpentine ulcers and cobblestoning). Additional characteristics include bowel stenosis or stricturing, as well as cobblestoning and linear ulcerations in the ileum [6].

IBD-U refers to cases where differentiating between UC and CD remains uncertain despite clinical, endoscopic, and pathological evaluations. Over time, some patients are reclassified, more often as CD than UC; however, the majority retain the diagnosis of IBD-U [7,8]. The terms IBD-U and indeterminate colitis are often used interchangeably. According to the Montreal Working Party, IBD-U is diagnosed based on clinical and endoscopic findings, including biopsies, whereas indeterminate colitis is reserved for colectomy specimens [9].

The Pediatric IBD (PIBD) Porto Group of the European Society for Paediatric Gastroenterology, Hepatology and Nutrition (ESPGHAN) developed a classification algorithm (PIBD classes criteria) to facilitate the standardized and objective classification of IBD subtypes in children [10]. The scoring system includes 23 features of IBD, many of which are histopathologic findings, categorized into three classes: features incompatible with UC (Class 1), features rarely found in UC (Class 2), and features uncommon in UC (Class 3). The algorithm classifies IBD subtypes based on the number of features present in each class. In 2020, a simplified version of the algorithm with 19 features was introduced and is considered reliable [11,12]. The classification further differentiates CD into CD and isolated colonic CD, reflecting the full spectrum of pediatric IBD. The simplified items of PIBD classes are listed in Table 1; for details on the modified classification algorithm, please refer to the article by Ledder et al. [11]. Moreover, efforts to refine pediatric IBD classification using machine learning have been reported, though many require further validation [13,14,15].

Pediatric IBD can also be classified by the patient’s age into the following categories: very-early-onset IBD (VEO-IBD), diagnosed before 6 years of age; infantile IBD, diagnosed before 2 years of age; and neonatal-onset IBD, diagnosed within the first 28 days of life [16]. An important consideration in diagnosing IBD in these younger age groups is the potential for monogenic causes (see the Section 5 for further details).

## 3. Upper Gastrointestinal Tract Involvement in Pediatric Inflammatory Bowel Disease

Upper endoscopy is routinely performed during the initial evaluation of suspected IBD in pediatric patients to provide a more comprehensive assessment, contrary to adult clinical practice where it is not typically recommended [17,18]. In addition to aiding disease classification, macro- and microscopic evaluations of the upper gastrointestinal tract are particularly useful in patients with isolated upper or small bowel disease and a normal colonoscopy.

Upper GI involvement is more common in patients with CD than in those with UC. Although any part of the upper GI tract may be affected, the stomach is the most frequently involved site in these patients [19]. The histologic examination plays a crucial role, as the microscopic disease can be detected even in patients with a macroscopically normal upper GI endoscopy [20]. In most cases, the inflammatory pattern is non-specific; however, several distinct pathologic features may be observed in IBD, including lymphocytic esophagitis, focally enhanced gastritis, Brunner gland lobulitis, and non-necrotizing granulomas at any site. Notably, aside from non-necrotizing granulomas, no pathologic findings are specific enough to differentiate CD from UC [18].

**Figure 1:**
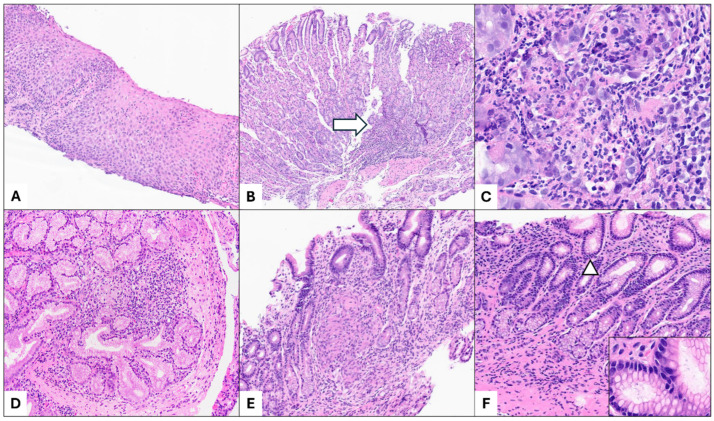
Upper gastrointestinal (GI) manifestations in pediatric inflammatory bowel disease (IBD) include (**A**) lymphocytic esophagitis, characterized by an increased number of intraepithelial lymphocytes with dilated intercellular spaces and parakeratosis (H&E, 4×). (**B**,**C**) Focally enhanced gastritis, marked by focal inflammation (white arrow) in the corpus mucosa, with neutrophilic infiltrates and associated epithelial injury (H&E, 4× and 20×). Notably, there is no significant background of chronic inflammation. (**D**) Brunner gland lobulitis in a patient with Crohn disease, showing focal acute inflammation involving mucosal Brunner glands (H&E, 10×). (**E**) Non-necrotizing granulomas, which most frequently affect the stomach in the upper GI tract, illustrated by antral mucosal involvement in a patient with Crohn disease (H&E, 10×). (**F**) Helicobacter infection should be ruled out in biopsies showing chronic active or inactive gastritis, as it may coexist with IBD (H&E, 10×). Microorganisms consistent with *Helicobacter pylori* are identified on H&E staining (white arrow and inset, H&E, 20×).

Lymphocytic esophagitis (LE) is characterized by esophageal mucosal injury (e.g., spongiosis and dyskeratotic cells) resulting from an increased number of intraepithelial lymphocytes in the absence of significant granulocytes (Figure 1A). The threshold for intraepithelial lymphocytosis remains somewhat arbitrary and debated, as data on the normal range of intraepithelial lymphocytes in the pediatric esophagus are lacking. In clinical practice, the authors adopt the diagnostic criteria proposed by Rubio et al. (i.e., >20 intraepithelial lymphocytes) [21]. A recent review by international experts suggested a higher threshold of >40 intraepithelial lymphocytes for diagnosis [22]. Additionally, the presence of epithelial injury is crucial to avoid overdiagnosis. LE has been reported in up to 28% of children with CD and 7% of those with UC [23,24]. In a recent cohort of children with untreated CD, Spasic et al. identified an association between LE and an increased need for anti-tumor necrosis factor therapy [25]. Beyond IBD, LE has been linked to various conditions, including motility disorders, rheumatologic conditions, hypothyroidism, and lichen planus [22,26]. Moreover, Candida infection, a frequent cause of esophageal intraepithelial lymphocytosis, should be carefully excluded during an evaluation [27,28].

Focally enhanced gastritis (FEG) is histologically defined by focal inflammatory infiltrates involving gastric glands, accompanied by epithelial injury. The infiltrates predominantly consist of lymphohistiocytic cells, with variable presence of neutrophils and eosinophils, within an otherwise unremarkable background (Figure 1B,C) [29]. Although FEG is not specific for IBD in adults, its positive predictive value for IBD in the pediatric population is relatively high (75%) [30,31]. This finding has been observed in both children with CD (54–55%) and UC (21–30%); therefore, it should not be used to differentiate between the two conditions [32,33]. FEG is most commonly found in the antral mucosa, with endoscopic correlates including aphthoid ulcers and submucosal bleeding [29]. Histologically, multifocal involvement may be present, with a higher total number of affected glands in UC compared to CD [32].

Brunner gland lobulitis has been proposed by Abdullgaffar et al. [34] as a specific finding in patients with CD. Histologically, it is a distinct form of duodenitis marked by focal acute inflammation involving the Brunner glands (Figure 1D). However, the literature on this pathologic finding is limited, particularly in the pediatric population. Based on the senior author’s experience, Brunner gland lobulitis can also be observed in children with a confirmed diagnosis of UC. Therefore, its specificity should be interpreted with caution until further studies provide a more definitive evidence.

Non-necrotizing granulomas can occur at any site within the upper GI tract in patients with CD, with the stomach being the most commonly affected, followed by the duodenum and esophagus [35]. In untreated patients, non-necrotizing granulomas are observed in more than half of the cases, whereas they are less frequently seen in treated patients [35,36]. In some patients, granulomas are found exclusively in the upper GI tract, which can aid in disease classification. Histologically, non-necrotizing granulomas consist of aggregates of epithelioid histiocytes, with or without multinucleated giant cells, and without evidence of caseation (Figure 1E) [18]. The differential diagnosis includes immunologic disorders, such as chronic granulomatous disease and common variable immune deficiency, as well as infections and granulomas associated with crypt epithelial injury (cryptolytic granulomas). The latter may necessitate an examination of multiple tissue levels to detect crypt rupture. Cryptolytic granulomas can be observed in any subtype of IBD; in pediatric UC, their presence in the upper GI tract has been associated with severe colonic involvement [37].

Additionally, there are several caveats to consider in the pathologic evaluation of upper GI biopsies performed in children with IBD. Although chronic inflammation, with or without acute inflammation, is commonly observed in the gastric mucosa of these patients, pathologists should carefully assess for the presence of *Helicobacter* infection. While earlier studies suggested an inverse association between *Helicobacter pylori* gastritis and IBD, raising the possibility that the infection may play a protective role against IBD development, a recent prospective multicenter study in a pediatric population found no significant difference in the prevalence of *Helicobacter pylori* gastritis between patients with IBD and healthy controls [38,39,40]. Helicobacter organisms can be identified on routine H&E staining (Figure 1F) or, when necessary, with immunohistochemical or special stains. Interestingly, pediatric patients with *Helicobacter* infection, even without an IBD diagnosis, may exhibit elevated fecal calprotectin levels, which can mimic IBD-related laboratory findings and potentially lead to unnecessary colonoscopies [41].

The duodenal mucosa in children with IBD may exhibit intraepithelial lymphocytosis (>25 intraepithelial lymphocytes per 100 enterocytes on routine H&E staining) with preserved villous architecture, resembling celiac disease (type 1 Marsh) [42]. A recent systematic review and meta-analysis has shown that there is an increased risk of IBD in patients with celiac disease and an increased risk of celiac disease in patients with IBD [43]. Moreover, the coexisting celiac disease has been reported more frequently in pediatric-onset IBD cases compared to adults [44]. Therefore, in pathology reports, it is prudent to include a note clarifying that while duodenal mucosa with intraepithelial lymphocytosis could represent an upper GI manifestation of IBD, celiac disease should be clinically ruled out. Similar pathologic findings may also be observed in *Helicobacter pylori* gastritis, hypersensitivity to non-gluten alimentary proteins, or medication effects [42].

## 4. Inflammatory Bowel Disease with Primary Sclerosing Cholangitis (PSC-IBD)

PSC-IBD has been proposed as a distinct entity from IBD not associated with PSC, with differences observed in genetics, intestinal and fecal microbiomes, and intestinal transcriptomics [45,46]. Similar to adults, PSC-IBD in the pediatric population demonstrates unique clinical and endoscopic features. Ricciuto et al. reported that endoscopic findings such as backwash ileitis, pancolitis, rectal sparing, and more severe right-sided disease activity are more prevalent in children with PSC-IBD compared to those with IBD not associated with PSC [47]. However, the absence of symptoms in these patients does not necessarily indicate a lack of mucosal inflammation. Additionally, children with PSC-IBD have demonstrated greater growth impairments compared to their counterparts with non-PSC-associated IBD. Recognizing this subtype is essential for patient management and prognostication. For pathologists, understanding the disease distribution and salient pathologic features is crucial, especially for early PSC recognition.

Histologically, PSC-IBD correlates well with endoscopic findings. The common pathological features include pancolitis, more severe right-colon colitis, backwash ileitis, lamina propria-predominant neutrophils, prominent lamina propria eosinophilia in the left colon, and surface villiform changes in the right colon [48]. However, in a large cohort of children with PSC-IBD, Little et al. reported a discordance between endoscopic and histologic rectal sparing, noting a similar frequency and low rates in both children with PSC-IBD and those with IBD not associated with PSC [48].

Does age at diagnosis influence PSC-IBD outcomes? Catassi et al. evaluated patients diagnosed with PSC-IBD before the age of 6 years (very-early-onset PSC-IBD) [49]. Despite similar baseline characteristics between younger and older children, very-early-onset PSC-IBD is associated with a higher frequency of PSC overlap with autoimmune hepatitis, and lower rates of biliary complications, including infective cholangitis and biliary strictures. At the other end of the spectrum, in adults, PSC-IBD is linked to an increased risk of colorectal dysplasia and cancer despite its typically mild clinical course [50,51]. Zhang et al. reported that dysplasia in PSC-IBD is often endoscopically invisible and pathologically non-conventional (e.g., crypt cell dysplasia, hypermucinous dysplasia, and goblet cell-deficient dysplasia) [51]. Due to these risks, current clinical guidelines recommend that patients with PSC-IBD undergo annual colonoscopies starting at the time of PSC diagnosis, regardless of the duration of IBD [52].

## 5. Monogenic Inflammatory Bowel Disease (IBD)

“Classic” IBD is polygenic and may be influenced by numerous factors. However, IBD in some patients can be attributed to alterations in a single gene; these cases are termed Monogenic IBD (mIBD). To be classified as having mIBD, a patient should present with signs and symptoms of IBD and have a rare, damaging gene variant that is known to cause a monogenic disorder from previous reports and is consistent with the patient’s clinical phenotype [53]. Typically, mIBD presents early in life and tends to have increased morbidity and mortality when compared to classic IBD, in part due to the associated co-morbidities.

The first cases of IBD deemed to be associated with a specific gene mutation were reported in 2009 by Glocker et al. [54]. They identified four cases with mutations in genes encoding the interleukin-10 receptor [54]. Since then, numerous other genes have been identified. The literature on mIBD is heterogenous and lacking in prospective studies. Each individual monogenic disorder has its own characteristics, making generalization difficult. In 2021, ESPGHAN established a consensus list of 75 genes implicated in mIBD, which in addition to clinical applications for diagnosis, has been utilized as a template by other groups carrying out research on mIBD [53,55].

Monogenic IBD is typically considered to be associated with VEO-IBD (IBD in a patient aged less than 6 years) [55]. However, patients may be diagnosed with mIBD throughout childhood and into adulthood, with the average age at diagnosis varying widely for individual genes; from less than 2 years for mIBD associated with SCID to the fourth decade in cases associated with GUCY2C mutations [53]. In one cohort of 1000 Canadian children with IBD, presumed monogenic causative mutations were identified in 7.8% of children younger than 6 years and 2.3% of children aged 6–18 years [56].

Consanguinity and family history of autoimmune disease or suspected/confirmed monogenic disorders are associated with monogenic IBD [55]. Genetics of mIBD typically follow Mendelian patterns of inheritance. However, some mIBD disorders, such as those involving LRBA or CTLA4, present with diverse phenotypes that complicate data acquisition for pedigrees and mask their inheritance patterns [55]. The majority of mIBD cases show autosomal recessive inheritance [53]. The next most frequent inheritance pattern is X-linked recessive, followed by autosomal dominant [53]. Heterozygous-like cases in the context of symptomatic female persons with one copy of genes typically associated with X-linked recessive inheritance and persons with one copy of a gene typically associated with autosomal recessive inheritance may also occur [53]. The family history of IBD is most common in heterozygous-like cases (47%), and least common in X-linked recessive (20%) [53]. Consanguinity may be seen in up to 35% of autosomal recessive cases [53].

The genes implicated in mIBD can be classified into broad categories based on gene function and clinical presentation. Nambu et al. identified 29 distinct monogenic disorders and seven common clinical presentations: autoimmunity, infection, hemophagocytic lymphohistiocytosis (HLH), hematolymphoid organ involvement, malignancy, dysmorphic features, and metabolic disease [53]. Many of the 29 monogenic disorders exhibit more than one of the clinical presentations (Table 2). Bolton et al. developed a taxonomy for the genes implicated in mIBD using syndromic features, response to hematopoietic stem cell transplantation (HSCT), RNA-sequencing, and proteomes of immune subsets, which in addition to classifying genes, may guide future genomic testing, an understanding of shared mechanisms, and directed use of therapies [57].

In some cases, the diagnosis may be aided by the recognition of certain histological patterns of inflammation in biopsy specimens. Wilkins et al. have proposed a pattern-based approach in evaluating patients with VEO-IBD which categorized the pathologic findings into five patterns: chronic active enteritis (conventional pathologic pattern in IBD), apoptosis/epithelial injury pattern, eosinophil-rich pattern, lymphocytic patterns, and granulomatous pattern (Table 3) [58].

Current treatments for mIBD may include surgery, small molecules, biologics, and/or HSCT. Bowel surgery was undertaken in 27% of the cases with rates of surgery ranging from 0 to 100% for individual genes [53]. Stoma creation occurred in 21% of surgeries and bowel resection in 63% [53]. Biologics were used in 33% of cases, with an efficacy rate of 25.5% [53]. HSCT has been carried out in 23% of cases with some degree of improvement in IBD symptoms in 72% of treated patients [53]. In some cases, gastrointestinal symptoms and colitis do not resolve after HSCT, despite remission of other symptoms, especially when epithelium is the primary site of disease, such as in NF-kappa B essential modulator or TTC7A deficiency [59]. Targeted therapies for specific genes have been proposed but are not currently widely accessible and show variable efficacy. Uhlig et al. have proposed a reporting system for therapeutic responses of individual patients in hopes of streamlining clinical care and supporting future research including meta-analysis [59].

The five genetic alterations most commonly implicated in mIBD involve interleukin-10 and its receptor (*IL-10/R*), X-linked inhibitor of apoptosis protein (*XIAP*), cytochrome b-245 beta chain (*CYBB*), LPS responsive beige-like anchor protein (LRBA), and tetratricopeptide repeat domain 7A (TTC7A) [53]. Together, these alterations contribute over 40% of the reported cases of mIBD [53].

### 5.1. IL-10R/R

Mutations in genes encoding the IL-10RA and B subunit proteins involve hyperinflammatory immune responses in the intestine [54]. Cases associated with IL-10/IL-10R account for over 15% of mIBD, more than any other group of genes [53]. Clinical findings are similar to those seen in polygenic CD such as enteric fistulas, perianal disease, abscesses, and chronic folliculitis [54,60]. Histological findings also largely mimic polygenic CD and include multifocal colonic ulcerations and collections of polymorphic infiltrates, with inflammation variably present in the small bowel [54,60]. Pear-shaped intramural abscesses extending into the submucosa and muscularis propria have also been reported [54]. Multimodal therapy, including some combination of exclusive enteral nutrition, anti-inflammatory and immunomodulatory agents may not achieve remission [60]. HSCT has also been shown to be effective [54].

### 5.2. XIAP

Similar to the other inhibitor of apoptosis genes, XIAP is a component of both innate and adaptive immunity [61]. Up to 9% of mIBD cases are associated with XIAP [53]. Cases may present with severe bloody diarrhea clinically within the first few weeks of life, refractory to conventional therapies [61]. Intestinal and extraintestinal manifestations may resemble CD with histology showing severe pancolitis with crypt abscesses and epithelium apoptosis [61,62]. A subset of patients with XIAP deficiency show early-onset colitis, severe failure to thrive, and splenomegaly/hepatosplenomegaly an increased risk of medically refractory IBD phenotype and increased mortality [62]. In this subset, early HSCT with reduced intensity conditioning may result in both resolution of their colitis and reduction in their risk of developing HLH, which is the leading cause of mortality in XIAP-deficient patients [62].

### 5.3. CYBB

**Figure 2:**
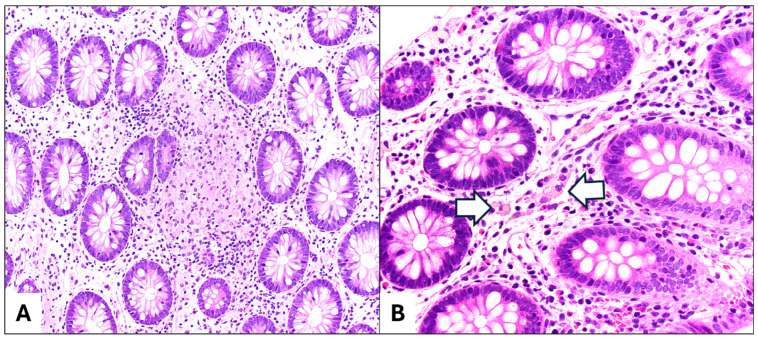
Chronic granulomatous disease is characterized by (**A**) an epithelioid granuloma in the lamina propria of the colonic mucosa (H&E, 10×) and (**B**) pigmented macrophages (white arrows; H&E, 20×). There is no significant active inflammation or chronicity in this colonic mucosa.

*CYBB* is one of the genes associated with Chronic Granulomatous Disease (CGD) [53]. *CYBB* alone is associated with 9% of mIBD cases [53]. Patients with CGD have a poor oxidative burst in their neutrophils resulting in the inability to clear certain infections and often present with a history of recurrent pneumonias, lymphadenopathy, upper GI involvement, and chronic colitis in early life [63]. Pathologic findings typically exhibit a granulomatous pattern and may also include mild chronic changes with increased lymphocytes and eosinophils in the lamina propria, and active colitis including crypt abscesses [63,64]. Granulomas in CGD are usually more confluent than in CD and may be accompanied by pigment-laden macrophages in the lamina propria (Figure 2A,B), which are thought to represent an accumulation of ceroid pigments due to the increased cell turnover [58]. The differentiation of CGD from CD is critical, because some IBD treatments, such as TNFα inhibitors, can lead to life-threatening infections in CGD patients [58]. A typical treatment of CGD includes subcutaneous interferon-gamma [63]. HSCT has shown good clinical responses including the normalization of oxidative burst and resolution of gastrointestinal symptoms [63,64]. Gene therapy with viral vectors has also been performed [59].

### 5.4. LRBA

Gene alterations in LRBA with resulting CTLA4 deficiency account for 4% of reported mIBD cases and fall under the umbrella of immunodysregulation polyendocrinopathy enteropathy X-linked (IPEX)-like syndrome [53]. They belong to the newly proposed functional category of “immune TOR-opathies” [65]. Clinically, the LRBA mutation may be associated with diarrhea, infections, and arthritis [58]. Histologically, a range of findings may be seen from a subtle lymphocytic pattern with surface and crypt intraepithelial lymphocytes and surface friability/denudation and normal mucosal architecture with slight increases in lamina propria cellularity to more classic patterns of chronic active colitis [58,65,66]. Targeted therapy directed to the mTOR pathway as well as individual genes with pathogenic variants has been proposed [65]. HSCT with reduced intensity conditioning has been used with good success [66].

### 5.5. TTC7A

Cases of mIBD associated with TTC7A represent another 4% of mIBD [53]. Patients with TTC7A mutations have disrupted intestinal and thymic epithelia and it was initially reported in children with multiple intestinal atresias and combined (T and B cell) immunodeficiency [67]. Small and large bowel biopsies in TTC7A-associated cases (with or without intestinal atresia) tend to show an epithelial injury-apoptosis pattern with predominantly neutrophilic infiltrates and numerous apoptotic bodies and diminished lymphocyte populations [58,67,68]. Additionally, atrophic gastritis has been described in a subset of patients [69]. HSCT in patients with TTC7A mutations may significantly increase their immune function, but as mentioned previously, it has not been shown to improve gastrointestinal disease [59,67]. Pharmacological targets in the PI4KIIIa-TTC7A-EFR3B pathway, with drugs such as leflunomide, represent a potential therapeutic approach [59,68].

## 6. Differential Diagnosis

Histologically, distinguishing IBD from its mimics remains a diagnostic challenge. A definitive diagnosis requires correlation with endoscopic findings, clinical history, and ancillary studies. Close communication between gastroenterologists and pathologists is essential and cannot be overemphasized. IBD should never be diagnosed solely on histologic grounds. When evaluating biopsies with a chronic active colitis pattern, two main alternative etiologies to consider are infection and medication effects. Other potential etiologies include vascular disorders, such as gastrointestinal manifestations of systemic vasculitis or drug-induced ischemia, as well as diversion proctocolitis, which affects nonfunctional bowel segments following surgery [70]. An accurate diagnosis is crucial, as treatment strategies often vary significantly depending on the underlying cause. A differential diagnosis for pattern-based injury (e.g., eosinophil-rich and apoptotic colitis) is summarized in Table 3.

Although infections typically present with an acute colitis pattern, some pathogens, such as *Salmonella* spp., *Shigella* spp., and *Entamoeba histolytica*, may exhibit features of chronicity that mimic IBD (e.g., architectural distortion, pyloric metaplasia, and Paneth cell hyperplasia) [71]. In older children and sexually active individuals, proctocolitis due to *Treponema pallidum* or *Chlamydia trachomatis* should also be considered, as these infections can closely resemble IBD both endoscopically and histologically [72]. However, the degree of chronicity in sexually transmitted infections is generally mild. Additionally, the presence of granulomas, particularly the necrotizing type, should prompt the consideration of infections caused by *Mycobacterium tuberculosis*, *Yersinia* spp., or *Actinomyces* spp. [71]. Stool cultures, PCR, and other clinical assays can aid in identifying infectious causes. In pediatric patients with infectious colitis, an underlying immunodeficiency should always be ruled out.

Drug-induced colitis can occur in all age groups, including pediatric patients, and presents with a wide spectrum of histologic patterns, ranging from active colitis and chronic active colitis (IBD-like) to microscopic colitis (lymphocytic/collagenous) and increased apoptosis [73]. Knowledge of the medication type, dosage, and the temporal relationship between drug exposure and symptom onset is critical for diagnosis. NSAIDs may induce a UC-like pattern with diffuse inflammation and ulceration or a CD-like pattern with granulomas [74]. Immune checkpoint inhibitors can cause acute colitis with or without chronicity, often with increased apoptotic bodies, a feature also seen in mycophenolate mofetil-associated colitis [73]. When an IBD-like injury pattern is present, the additional findings of intraepithelial lymphocytosis or prominent apoptosis should alert the pathologist to the possibility of drug-induced colitis.

## 7. Conclusions

In the pediatric population, the diagnosis and classification of IBD require an understanding of both classical and atypical presentations. Upper gastrointestinal manifestations are frequently observed in pediatric IBD, which is likely due to the routine inclusion of upper endoscopy in the diagnostic workup. Histopathological findings may reveal a non-specific inflammation; however, certain distinct patterns, such as LE, FEG, Brunner gland lobulitis, and non-necrotizing granulomas, should prompt the consideration of an IBD diagnosis. PSC-IBD and mIBD, though not exclusive to the pediatric age group, represent specific subtypes with important implications for prognosis and management. Early recognition of the clinical and pathological features of PSC-IBD can facilitate the timely detection of PSC. While PSC-IBD is generally associated with a milder disease course, it carries an increased risk for colorectal dysplasia and carcinoma. Furthermore, factors such as the patient’s age, family history, consanguinity, and comorbidities should be carefully considered to identify potential monogenic causes of IBD.

## Figures and Tables

**Table 1:** Simplified items of PIBD classes proposed by Ledder et al. [11] (histologic criteria in bold italics). This table is reproduced from [11] with permission from Oxford University Press. It is not subject to the CC BY license of this article. For further use, please refer to the original publisher’s rights and permissions.

**Class 1** (features incompatible with UC) ***1.*** ***At least one well-formed granuloma anywhere in the GI tract (cryptolytic granulomas excluded);*** 2. At least one of the following: deep ulcerations; cobblestoning; or stenosis anywhere in the small bowel or upper GI tract (stomach excluded); 3. Fistulizing disease; 4. Thickened jejunal or ileal bowel loops on imaging/significant small bowel inflammation on capsule endoscopy (backwash ileitis excluded); ***5.*** ***Any ileal inflammation in the presence of normal cecum (incompatible with backwash ileitis);***
**Class 2** (features rarely found in UC) ***6.*** ***Macro- and microscopically normal appearing skip lesions in untreated patients (rectal sparing and cecal patch excluded);*** ***7.*** ***Complete rectal sparing (macroscopic and microscopic);*** ***8.*** ***Macroscopically normal colon between the inflamed mucosa, but with microscopic inflammation (relative patchiness);*** 9. Significant growth delay (height velocity < 2SD), not explained by other causes; ***10.*** ***Transmural inflammation of the colon in the absence of severe colitis;*** 11. Presence of any small and not deep ulcers in the small bowel and esophagus, or >5 small and not deep ulcers in the stomach or colon, with background normal mucosa, not explained by other causes; 12. Positive ASCA in the presence of negative pANCA; ***13.*** ***Reverse gradient of mucosal inflammation (proximal > distal; rectal sparing excluded);*** 14. Deep ulcerations or severe cobblestoning of the stomach or scalloping of the duodenum, not explained by other causes;
**Class 3** (features uncommon in UC) ***15.*** ***Focal chronic duodenitis on histology;*** 16. ***Focal active colitis on histology in more than one biopsy;*** 17. Several (<5) aphthous ulcerations in the colon or in the stomach; 18. Non-bloody diarrhea; ***19.*** ***Focally enhanced gastritis on histology.***

SD: standard deviation; ASCA: anti-Saccharomyces cerevisiae antibody; pANCA: perinuclear anti-neutrophil cytoplasmic antibody.

**Table 2:** Genotypes and phenotypes in monogenic forms of IBD.

Monogenic Disorder	Genes	IBD Type Reported	Extraintestinal Associations (in Order of Relative Frequency)
Agammaglobulinemia	*BTK*, *PIK3CD*, *PIK3R1*	CD/CD-like, IBD, UC/UC-like	Autoimmunity, infection
Chronic enteropathy associated with *SLCO2A1* gene	*SLCO2A1*	IBD, CD/CD-like	Autoimmunity
Chronic granulomatous disease	*CYBA*, *CYBB*, *NCF1*, *NCF2*, *NCF4*	Granulomatous colitis, IBD, CD/CD-like, UC/UC-like, IBD-U/IC	Autoimmunity, infection, HLH, malignancy
CHAPLE syndrome	*CD55*	ND	Autoimmunity, infection, malignancy
DOCK8 syndrome	*DOCK8*	UC/UC-like, CD/CD-like, IBD	Autoimmunity, infection
Familial hemophagocytic lymphohistiocytosis 4	*STXBP2*	ND	Autoimmunity, infection, HLH
Glycogen storage disease type 1B	*SLC37A4*	IBD, CD/CD-like	Infection, hematolymphoid involvement, metabolic disease
Familial GUCY2C diarrhea syndrome	*GUCY2C*	CD/CD-like, IBD	
G6PC3 deficiency	*G6PC3*	CD/CD-like, IBD	Infection, dysmorphic features
Haploinsufficiency of A20	*TNFAIP3*	Intestinal BD, CD/CD-like, IBD	Autoimmunity, infection
Hermansky-Pudlak syndrome	*HPS1*, *HPS4*, *HPS6*	CD/CD-like, IBD, UC/UC-like	Autoimmunity
Hoyeraal-Hreidarsson Syndrome	*DKC1*, *RTEL1*	UC/UC-like, CD/CD-like	Dysmorphic features, infection
IL-10 and IL-10-receptor associated colitis	*IL-10*, *IL-10RA*, *IL-10RB*	IBD, CD/CD-like, IBD-U/IC	Autoimmunity, infection, HLH
Immunodysregulation polyendocrinopathy enteropathy X-linked syndrome (IPEX)	*FOXP3*	IBD, CD/CD-like, UC/UC-like, IBD-U/IC	Autoimmunity, infection
Immunodysregulation polyendocrinopathy enteropathy X-linked-like syndrome (IPEX-like)	*STAT1*, *STAT3*, *CTLA4*, *LRBA*, *IL21*, *IL2RA*, *IL2RB*, *MALT1*	IBD, CD/CD-like, UC/UC-like, IBD-U	Autoimmunity, infection, malignancy, hematolymphoid involvement
Kindler syndrome	*FERMT1*	UC/UC-like, CD/CD-like	
Leukocyte adhesion deficiency	*ITGB2*	ND	Infection
Loeys-Dietz syndrome	*TGFBR1*, *TGFBR2*	UC/UC-like, CD/CD-like, IBD	Dysmorphic features, infection
Mevalonate kinase deficiency	*MVK*	ND	Autoimmunity
Nuclear factor-kappa B Essential Modulator deficiency (NEMO)	*IKBKG*	IBD, CD/CD-like, intestinal BD	Dysmorphic features, infection
NLRC4-macrophage activating syndrome	*NLRC4*	ND	Autoimmunity, infection, HLH
Niemann-Pick disease type C	*NPC1*	CD/CD-like, IBD-U/IC	Metabolic disease
RIPK1 deficiency	*RIPK1*	IBD	Autoimmunity, infection
Severe combined immunodeficiency	*CD3G*, *DCLRE1C*, *IL2RG*, *LIG4*, *RAG1*	IBD, CD/CD-like	Infection
Trichohepatoenteric syndrome	*SKIV2L*, *TTC37*	IBD-U/IC, IBD, CD/CD-like	Dysmorphic features, infection
TTC7A deficiency	*TTC7A*	IBD, IBD-U/IC, CD/CD-like	Autoimmunity, infection, dysmorphic features, malignancy
TRIM22 deficiency	*TRIM22*	ND	Autoimmunity, infection
Wiskott-Aldrich syndrome/Wiskott-Aldrich-like syndrome (WAS/WAS-like)	*WAS*, *ARPC1B*	IBD, CD/CD-like, UC/UC-like	Autoimmunity, infection
XIAP deficiency	*XIAP*	CD/CD-like, IBD, UC/UC-like, IBD-U/IC	Autoimmunity, infection, HLH, hematolymphoid involvement, malignancy

CD: Crohn disease; IBD: inflammatory bowel disease; UC: ulcerative colitis; IBD-U: inflammatory bowel disease-unclassified; IC: indeterminate colitis; ND: not described; HLH: hemophagocytic lymphohistiocytosis.

**Table 3:** Histologic patterns and underlying causes of very-early-onset IBD.

Pattern	Key Findings	Associated Monogenic Disorders	Other Causes of Pattern
Chronic active enteritis	Active inflammation (neutrophilic cryptitis and crypt abscesses) Chronic mucosal changes (architectural changes and cellular metaplasia)	IL-10/R; CGD; XIAP; GUCY2C; familial Mediterranean fever; HPS types 1, 4, 6; GSD1; WAS; CVID; TTC7A	Polygenic IBD
Apoptosis/Epithelial injury pattern	Epithelial injury or apoptosis out of proportion to inflammatory activity	Disorders resulting in B and T cell deficiency/dysregulation; SCID; Omenn syndrome; IPEX; TTC7A; dystrophic epidermolysis bullosa; mevalonate kinase deficiency; disorders of telomere maintenance	GvHD; antimetabolite immunosuppression, especially mycophenolate mofetil; acute GI transplant rejection; viral infection
Eosinophil-rich pattern	Clusters of eosinophils in the lamina propria and eosinophilic infiltration of surface and crypt epithelium	Omenn syndrome	Non-monogenic IBD; non-IBD etiologies for GI eosinophilia (e.g., allergy, parasitic infection, Langerhans cell histiocytosis)
Lymphocytic patterns	Lymphocytosis and nodular lymphoid hyperplasia OR Lymphocyte or plasma cell depletion AND Lack of active inflammation and chronic mucosal changes	Lymphocytosis: CVID; IgA deficiency; CTLA4-related disorders; T cell regulatory disorders; IPEX Lymphocyte or plasma cell depletion: CVID; hypogammaglobulinemia; X-linked lymphoproliferative disorder type 1; SCID	Lymphocytosis: celiac disease; post-infectious enteritis (can be secondary to immunodeficiency), medication effect (especially NSAIDs); bacterial overgrowth Lymphoid depletion: medical therapy (e.g., conditioning chemotherapy prior to bone marrow transplant); artifactual due to mucosal edema
Granulomatous pattern	Non-necrotizing granulomas	CGD; IL-10/R; HPS types 1, 4, 6	Misidentification of cryptolytic granuloma/germinal center; infectious granulomas (mycobacterial/fungal, usually necrotizing); or very rarely sarcoidosis

IL-10/R: IL-10 and IL-10-receptor associated colitis; CGD: chronic granulomatous disease; XIAP: X-linked inhibitor of apoptosis protein; GUCY2C: familial *GUCY2C* diarrhea syndrome; HPS: Hermansky-Pudlak syndrome; GSD1: glycogen storage disease type 1; WAS: Wiskott-Aldrich syndrome; CVID: common variable immune deficiency; SCID: severe combined immunodeficiency; IPEX: immunodysregulation polyendocrinopathy enteropathy X-linked syndrome; IBD: inflammatory bowel disease; GvHD: graft versus host disease; NSAIDs: non-steroidal anti-inflammatory drugs.
